# From Phenomenon to Essence: A Newly Involved lncRNA Kcnq1ot1 Protective Mechanism of Bone Marrow Mesenchymal Stromal Cells in Liver Cirrhosis

**DOI:** 10.1002/advs.202206758

**Published:** 2023-06-06

**Authors:** Hanjing Zhangdi, Yanan Jiang, Yang Gao, Shuang Li, Ruiling Xu, Jing Shao, Jingyang Liu, Ying Hu, Xu Zhang, Xiaoyu Zhang, Lei Zhao, Jihan Qi, Xinyu Geng, Shizhu Jin

**Affiliations:** ^1^ Department of Gastroenterology and Hepatology The Second Affiliated Hospital of Harbin Medical University Harbin 150001 China; ^2^ Department of Pharmacology (State‐Province Key Laboratories of Biomedicine‐Pharmaceutics of China, Key Laboratory of Cardiovascular Research, Ministry of Education) College of Pharmacy Harbin Medical University Harbin 150081 China; ^3^ Translational Medicine Research and Cooperation Center of Northern China Heilongjiang Academy of Medical Sciences Harbin 150081 China

**Keywords:** bone marrow mesenchymal stem cells, LncRNA Kcnq1ot1, liver cirrhosis, miR‐374‐3p, Fstl1, Creb3l1

## Abstract

Bone marrow mesenchymal stromal cells (BMSCs) have a protective effect against liver cirrhosis. Long noncoding RNAs (lncRNAs) play crucial roles in the progression of liver cirrhosis. Therefore, it is aimed to clarify the lncRNA Kcnq1ot1 involved protective mechanism of BMSCs in liver cirrhosis. This study found that BMSCs treatment attenuates CCl_4_‐induced liver cirrhosis in mice. Additionally, the expression of lncRNA Kcnq1ot1 is upregulated in human and mouse liver cirrhosis tissues, in addition to TGF‐*β*1‐treated LX2 cells and JS1 cells. The expression of Kcnq1ot1 in liver cirrhosis is reversed with BMSCs treatment. The knockdown of Kcnq1ot1 alleviated liver cirrhosis both in vivo and in vitro. Fluorescence in situ hybridization (FISH) confirms that Kcnq1ot1 is mainly distributed in the cytoplasm of JS1 cells. It is predicted that miR‐374‐3p can directly bind with lncRNA Kcnq1ot1 and Fstl1, which is verified via luciferase activity assay. The inhibition of miR‐374‐3p or the overexpression of Fstl1 can attenuate the effect of Kcnq1ot1 knockdown. In addition, the transcription factor Creb3l1 is upregulated during JS1 cells activation. Moreover, Creb3l1 can directly bind to the Kcnq1ot1 promoter and positively regulate its transcription. In conclusion, BMSCs alleviate liver cirrhosis by modulating the Creb3l1/lncRNA Kcnq1ot1/miR‐374‐3p/Fstl1 signaling pathway.

## Graphical abstract

BMSCs alleviate liver cirrhosis by modulating the Creb3l1/lncRNA Kcnq1ot1/miR–374–3p/Fstl1 signaling pathway. This research offers a novel theoretical framework for the use of BMSCs in the treatment of liver cirrhosis, with the potential to influence in future clinical diagnosis and therapy, which may promote the clinical application of BMSCs.

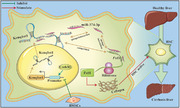

## 1 Introduction

Globally, liver cirrhosis is a life‐threatening disease, as it can develop into liver failure or even hepatocellular carcinoma.^[^ ^1^ ^]^ In the past two decades, the number of liver cirrhosis patients worldwide increased by 74.5%.^[^ ^2^ ^]^ Liver fibrosis is an early stage of liver cirrhosis, which is characterized by the activation of hepatic stellate cells (HSCs) and the deposition of the extracellular matrix. With the progression of liver fibrosis, pseudolobules gradually form, and cirrhosis occurs.^[^ ^3^ ^]^ Currently, there is no clinically appropriate treatment for advanced liver cirrhosis. Fortunately, the application of bone marrow mesenchymal stromal cells (BMSCs) therapy brings new insight to the treatment of cirrhosis.

BMSCs are derived from mammalian bone marrow, and their low immunogenic properties make them useful for autologous or allogeneic cell transplantation therapy.^[^ ^4^ ^]^ BMSCs therapy has already been shown effective for liver cirrhosis.^[^ ^5^ ^]^ Previous studies have shown that BMSCs could alleviate liver cirrhosis through multiple pathways, including homing,^[^ ^6^ ^]^ differentiation into hepatocyte‐like stem cells,^[^ ^7^ ^]^ paracrine hepatocyte growth factor execution,^[^ ^8^ ^]^ and TGF‐*β*/SMAD pathway inhibition.^[^ ^9^ ^]^ etc. The mechanism of BMSCs in the treating liver cirrhosis is being continuously investigated. A recent study showed that BMSCs could alleviate liver fibrosis by regulating long noncoding RNA (lncRNA) BIHAA1 in HSCs, indicating that BMSCs may contribute to the treatment of liver cirrhosis through the regulation of lncRNAs.^[^ ^10^ ^]^

LncRNAs are defined as a class of RNA transcripts longer than 200 nucleotides with diverse biological functions.^[^ ^11^, ^12^ ^]^ Studies showed that BMSCs played a therapeutic role by regulating the expression of lncRNAs in various diseases. Liu et al.^[^ ^13^ ^]^ found that the BMSC‐derived exosomal lncRNA PTENP1 suppressed bladder cancer progression. Han et al.^[^ ^14^ ^]^ demonstrated that lncRNA KLF3‐AS1 induced angiogenesis in diabetic skin, promoting wound healing. In addition, many effective lncRNAs have been identified as promising targets for diagnosing and treating liver disease.^[^ ^15^ ^]^ Our previous study found that 374 lncRNAs were differentially expressed in cirrhosis, among which, lncRNA Kcnq1ot1 was highly expressed.^[^ ^16^ ^]^ Moreover, studies have found that Kcnq1ot1 is upregulated in liver fibrosis, and the inhibition of Kcnq1ot1 expression has an anti‐fibrosis effect.^[^ ^17^ ^]^ However, whether lncRNA Kcnq1ot1 is involved in BMSC therapy for liver cirrhosis remains unknown.

In our preliminary experiment, BMSCs were found to inhibit Kcnq1ot1 expression. Therefore, in this study, we aimed to determine the lncRNA Kcnq1ot1‐based protective mechanism of BMSCs in liver cirrhosis.

## 2 Results

### 2.1 BMSCs Alleviate Liver Cirrhosis Both In Vivo and In Vitro.

**Figure 1:**
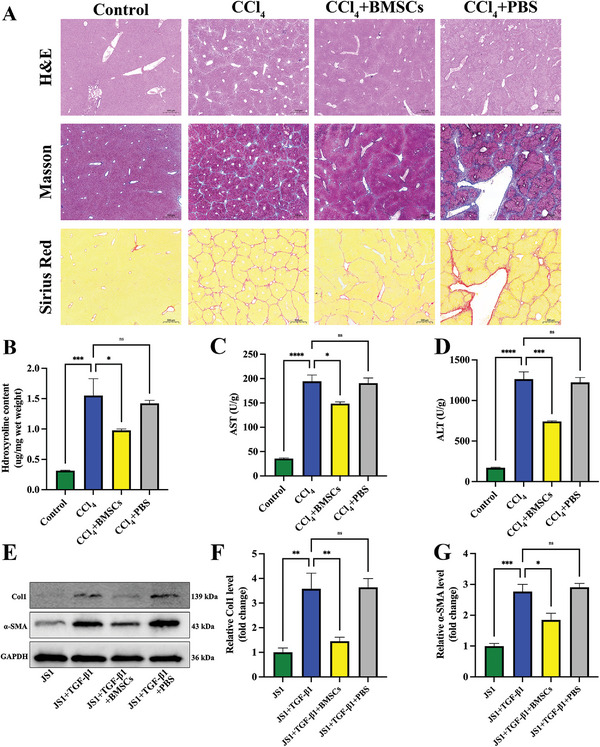
BMSCs alleviate liver cirrhosis in vivo and prevent HSCs activation in vitro. A) H&E, Masson and Siris Red staining images. B) Liver hydroxyproline content. C,D) Liver AST (C) and ALT (D) expression levels. E) Representative western blot bands for Col1 and *α*‐SMA in JS1 cells. F,G) Statistical results of relative Col1 (F) and *α*‐SMA (G) expression levels. *n* = 3 in each group. * *p* < 0.05, ** *p* < 0.01, ***, *p* < 0.001, ns *p* ≥ 0.05.

We established liver cirrhosis models and treated them using BMSCs both in vivo and in vitro (Figure S1A,B, Supporting Information). We verified the primary BMSCs by flow cytometry according to our previous experimental method.^[^ ^18^ ^]^ The positive expression of CD29 and CD90 indicated that the extracted cells were BMSCs. The negative expression of CD31 and CD43 excluded the presence of other cells (Figure S1C, Supporting Information). To confirm the differentiation capacity of the primary isolated BMSCs, we used alizarin red to label osteoblast‐induced calcium nodules and oil red O to label lipid‐induced lipid droplets (Figure S1D, Supporting Information). BMSCs with DiR‐labeled cell membranes also mainly appeared in cirrhotic liver tissue (Figure S1E, Supporting Information). H&E, Masson, and Sirius red pathological staining also showed severe cirrhosis, with pseudolobule formations in the liver cirrhosis group, while BMSCs treatment attenuated the progression of liver cirrhosis (**Figure** **1**A). Here, hydroxyproline quantitative analysis reflected the degree of cirrhosis. It was found that BMSCs treatment decreased hydroxyproline content in liver tissues (Figure 1B). Further, the liver function indicators AST (aspartate aminotransferase) and ALT (alanine aminotransferase) were upregulated in liver cirrhosis tissues, which BMSCs treatment alleviates (Figure 1C,D). When liver cirrhosis occurs, hypoxia‐induced growth factors can promote intrahepatic bile duct hyperplasia, the CK19‐labeled intrahepatic bile duct can indirectly reflect the degree of liver cirrhosis, and BMSCs treatment can inhibit intrahepatic bile duct hyperplasia (Figure S2A, Supporting Information). Simultaneously, BMSCs treatment downregulated the *α*‐SMA and Col1 (Collagen 1) protein expression levels both in vivo (Figure S2B, Supporting Information) and in vitro (Figure 1E–G).

### 2.2 LncRNA Kcnq1ot1 is Upregulated in Liver Cirrhosis Tissues and Activated Hepatic Stellate Cells

**Figure 2:**
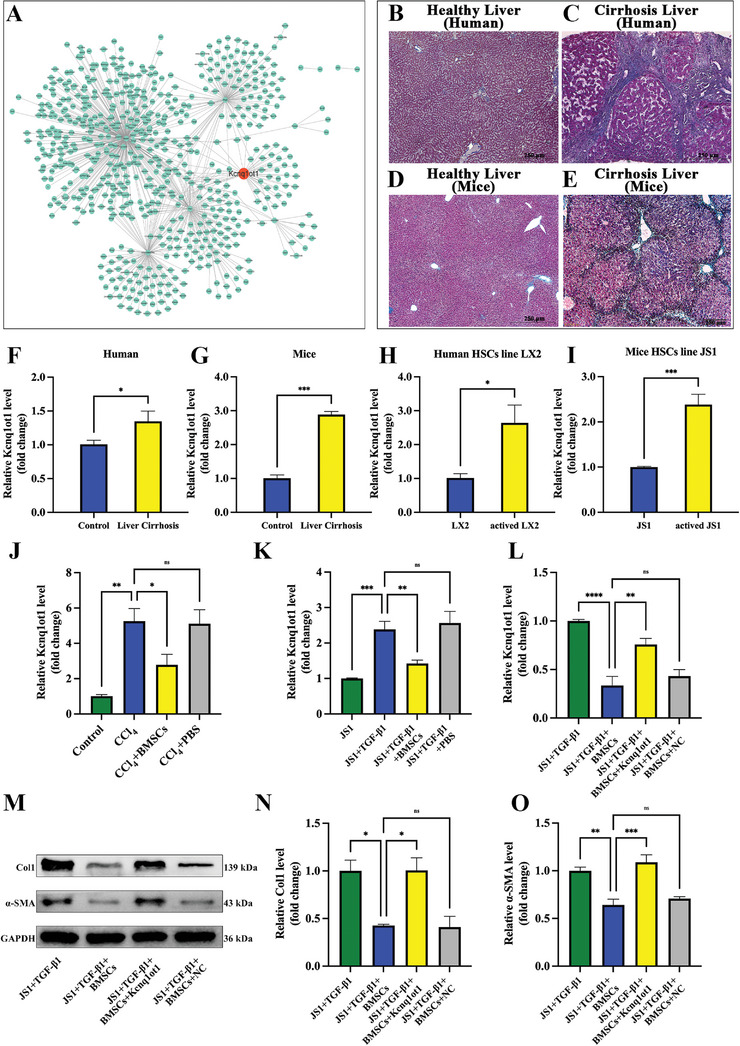
Kcnq1ot1 is elevated in vivo and vitro in cirrhosis and can be inhibited by BMSC administration. A) The interaction networks of differentially expressed lncRNAs and mRNAs in cirrhosis. B,C) Masson staining of human healthy liver (B) and cirrhotic liver tissues (C). D,E) Masson staining of mouse healthy liver (D) and cirrhotic liver tissues (E). F–I) LncRNA Kcnq1ot1 expression in human (F) and mouse tissues (G) and in LX2 (H) and JS1 (I) cells. J,K) The effect of BMSC treatment on lncRNA Kcnq1ot1 expression in cirrhotic liver (J) and activated JS1 cells (K). L) Overexpression of lncRNA Kcnq1ot1 can alleviate the inhibitory effect of BMSCs on Kcnq1ot1 in activated JS1 cells. M) Representative western blot bands of Col1 and *α*‐SMA in JS1 cells. N,O) Statistical results of relative Col1 (N) and *α*‐SMA (O) expression level. *n* = 3 in each group. * *p* < 0.05, ** *p* < 0.01, *** *p* <0.001, **** *p* <0.0001, ns *p* ≥0.05.

Based on our previous RNA sequencing data,^[^ ^16^ ^]^ a lncRNA‐mRNA interaction network was constructed using differentially expressed genes. The constructed network contained 1186 nodes (988 mRNAs, 146 lncRNAs, and 52 miRNAs) and 1838 edges. Among them, Kcnq1ot1 was one of the hub nodes and was elevated in cirrhosis (**Figure** **2**A). Here, Masson staining demonstrated that cirrhosis was induced by CCl_4_ (Figure 2B–E). Kcnq1ot1 expression was also elevated in human and mouse liver cirrhosis (Figure 2F,G). Besides, Kcnq1ot1 expression was also increased in human and mice activated HSC cell lines (Figure 2H,I).

### 2.3 BMSCs Relieve Liver Cirrhosis by Downregulating Kcnq1ot1 Expression

The expression of Kcnq1ot1 was decreased after BMSCs treatment in mice with liver cirrhosis (Figure 2J). Similarly, BMSCs treatment inhibited Kcnq1ot1 expression in TGF‐*β*1‐treated JS1 cells (Figure 2K). In addition, overexpression of Kcnq1ot1 in activated JS1 cells can reverse the regulatory effect of BMSCs on Kcnq1ot1 (Figure 2L). We then detected the alteration of fibrosis markers. BMSCs treatment inhibited *α*‐SMA and Col1 protein expression levels in TGF‐*β*1‐treated JS1 cells, while the overexpression of Kcnq1ot1 alleviated the protective effect of BMSCs (Figure 2M–O).

### 2.4 The Silencing of Kcnq1ot1 Suppresses the Proliferation and Activation of JS1 Cells

**Figure 3:**
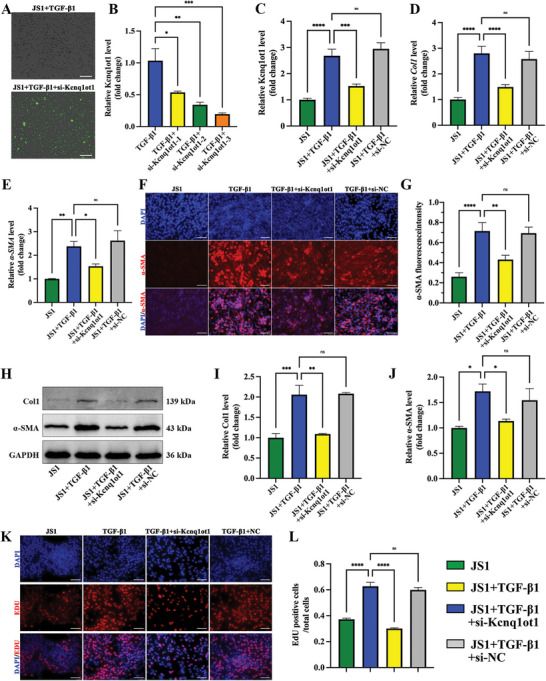
Silencing of lncRNA Kcnq1ot1 suppresses the proliferation and activation of JS1 cells. A) The transfection efficiency of siRNA in JS1 cells. The GFP fluorescence was detected in 488 nm. B) The knockdown efficiency of 3 siRNA sequences targeting lncRNA Kcnq1ot1. C) LncRNA Kcnq1ot1 expression in JS1 cells. D‐E) Col1 (D) and *α*‐SMA (E) mRNA expression in JS1 cells. F) Immunofluorescence images of *α*‐SMA in JS1 cells. G) The fluorescence intensity of *α*‐SMA in JS1 cells. H) Representative western blot bands of Col1 and *α*‐SMA in JS1 cells. I‐J) Statistical results of Col1 (I) and *α*‐SMA (J) expression level. K‐L) EdU staining images (K) and proliferation (L) of JS1 cells by si‐Kcnq1ot1 transfection. *n* = 3 in each group. * *p* < 0.05, ** *p* < 0.01, *** *p* < 0.001, **** *p* < 0.0001, ns *p* ≥ 0.05. Scale bar, 50 µm.

The transfection efficiency of siRNA in JS1 cells was reflected by the fluorescence of GFP (**Figure** **3**A). Three siRNA sequences were designed to knockdown Kcnq1ot1. The knockdown effect of si‐Kcnq1ot1‐3 was higher than the other two and was used in the following experiments (Figure 3B). The expression of Kcnq1ot1 was also elevated in activated JS1 cells, which was downregulated by the transfection of si‐Kcnq1ot1 (Figure 3C). Furthermore, the qRT‐PCR, western blot assay, and immunofluorescence staining showed that the activation of JS1 cells promoted *α*‐SMA and Col1 expression at both mRNA and protein levels. Therefore, the knockdown of lncRNA Kcnq1ot1 can alleviate the activation of JS1 cells (Figure 3 D–J). In addition, EdU staining verified that the cell proliferation ability of activated JS1 cells was inhibited after Kcnq1ot1 knockdown (Figure 3K,L).

### 2.5 LncRNA Kcnq1ot1 Directly Binds with miR‐374‐3p

**Figure 4:**
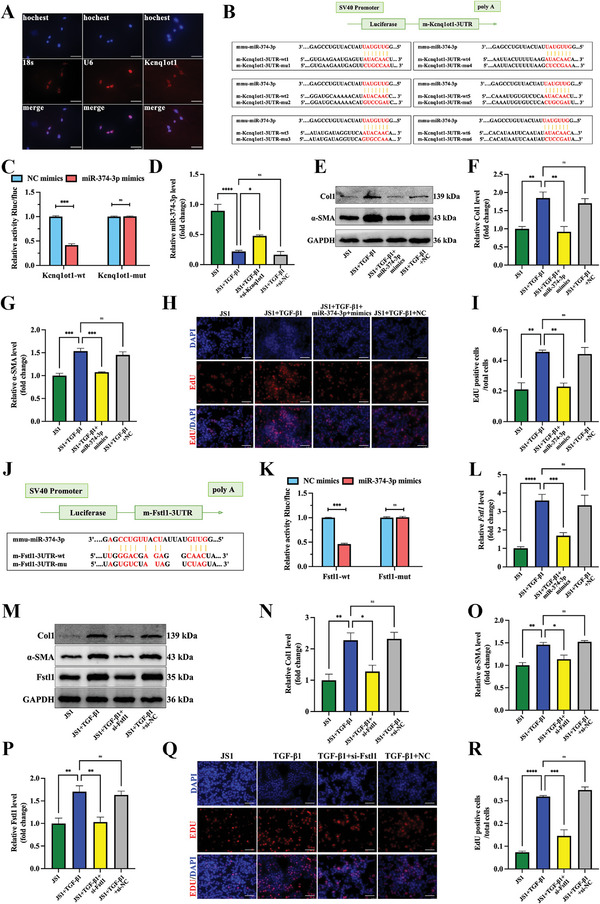
miR‐374‐3p directly binding with lncRNA Kcnq1ot1 and Fstl1. A) Distribution of lncRNA Kcnq1ot1 in JS1 cells by FISH. B) The predicted binding sites between miR‐374‐3p and lncRNA Kcnq1ot1. C) Luciferase activity assay showed the direct interaction between lncRNA Kcnq1ot1 and miR‐374‐3p. D) MiR‐374‐3p expression in JS1 cells. E) Representative western blot bands of Col1 and *α*‐SMA in JS1 cells. F‐G) Statistical results of Col1 (F) and *α*‐SMA (G) expression level. H,I) EdU staining images (H) and proliferation (I) of JS1 cells by miR‐374‐3p mimic transfection. J) The predicted binding sites between miR‐374‐3p and Fstl1. K) Luciferase activity assay showed the direct interaction between miR‐374‐3p and Fstl1. L) Relative Fstl1 mRNA expression level in JS1 cells. M) Representative western blot bands of Col1, *α*‐SMA and Fstl1in JS1 cells. N–P) Statistical results of Col1 (N), *α*‐SMA (O), and Fstl1 (P) expression level. Q‐R) EdU staining images (Q) and proliferation (R) of JS1 cells by si‐Fstl1 transfection. *n* = 3 in each group, * *p* < 0.05, ** *p* < 0.01, *** *p* < 0.001, **** *p* < 0.0001, ns *p* ≥ 0.05. Scale bar, 50 µm.

We then explored the distribution of lncRNA Kcnq1ot1 in JS1 cells using fluorescence in situ hybridization (FISH) and found that Kcnq1ot1 is mainly localized in the cytoplasm (**Figure** **4**A). The binding site between Kcnq1ot1 and miR‐374‐3p was predicted using the online software starBase 3.0 (Figure 4B). The luciferase activity assay verified the relationship between Kcnq1ot1 and miR‐374‐3p. The results showed that a mimic of miR‐374‐3p downregulated the luciferase activity of wide type (wt) Kcnq1ot1, with little effect on mutation (mut) Kcnq1ot1 (Figure 4C). The expression of miR‐374‐3p was also decreased in activated JS1 cells but increased after the silencing of Kcnq1ot1 (Figure 4D). *α*‐SMA and Col1 expression were downregulated by transfection of miR‐374‐3p mimics (Figure 4E–G). Finally, the proliferation of activated JS1 cells was increased, which was inhibited by the enhanced expression of miR‐374‐3p (Figure 4H,I).

### 2.6 Fstl1 is a Direct Target of miR‐374‐3p

The binding site between miR‐374‐3p and Fstl1 was predicted using starBase 3.0 (Figure 4J) and confirmed it using a luciferase activity assay. The results revealed that the overexpression of miR‐374‐3p suppressed the luciferase activity of WT Fstl1 while exerted no effect on MUT Fstl1 (Figure 4K). Nevertheless, the miR‐374‐3p mimic downregulated the Fstl1 mRNA expression level (Figure 4L). *α*‐SMA, Col1, and Fstl1 protein expression were also downregulated by si‐Fstl1 in activated JS1 cells (Figure 4M–P). Finally, the proliferation of JS1 cells was inhibited by si‐Fstl1 (Figure 4Q–R).

### 2.7 LncRNA Kcnq1ot1 Regulates JS1 Cells Activation via the miR‐374‐3p/ Fstl1 Signaling Pathway

**Figure 5:**
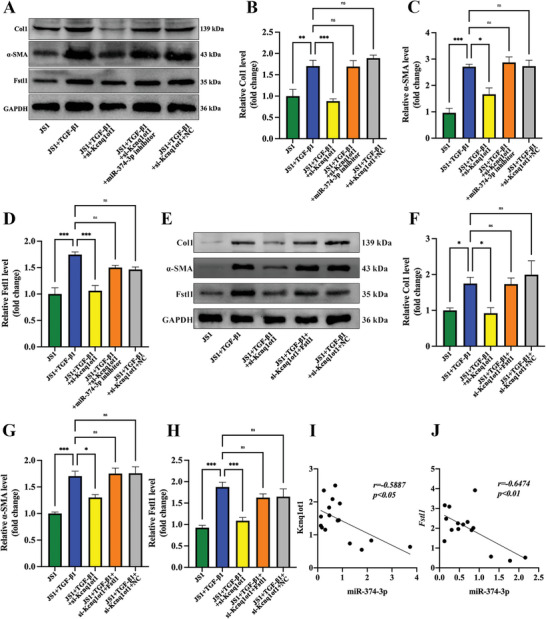
LncRNA Kcnq1ot1 regulates JS1 cells activation via miR‐374‐3p/Fstl1 pathway. A) Representative western blot bands of Col1, *α*‐SMA, and Fstl1 in JS1 cells with si‐Kcnq1ot1 and miR‐374‐3p inhibitor. B–D) Statistical results of Col1 (B), *α*‐SMA (C), and Fstl1 (D) expression level in JS1 cells with si‐Kcnq1ot1 and miR‐374‐3p inhibitor. E) Representative western blot bands of Col1, *α*‐SMA, and Fstl1 in JS1 cells with si‐Kcnq1ot1 and Fstl1 overexpression. F—H) Statistical results of Col1 (F), *α*‐SMA (G), and Fstl1 (H) expression level in JS1 cells with si‐Kcnq1ot1 and Fstl1 overexpression. I) Relationship between lncRNA Kcnq1ot1 and miR‐374‐3p by Pearson correlation analysis. J) Relationship between miR‐374‐3p and Fstl1 by Pearson correlation analysis. *n* = 3 in each group, * *p* < 0.05, ** *p* < 0.01, *** *p* < 0.001, **** *p* < 0.0001, ns *p* ≥ 0.05.

To investigate the interaction among lncRNA Kcnq1ot1, miR‐374‐3p, and Fstl1, Kcnq1ot1 and miR‐374‐3p were knocked down in activated JS1 cells. The results showed that the knockdown of Kcnq1ot inhibited *α*‐SMA, Col1, and Fstl1 expression, which was reversed by co‐transfected with miR‐374‐3p inhibitor (**Figure** **5**A–D). In addition, the overexpression of Fstl1 could also inhibit the effect of si‐Kcnq1ot1 on the protein expression of *α*‐SMA, Col1, and Fstl1 in activated JS1 cells (Figure 5E–H). Statistically, miR‐374‐3p was negatively correlated with Kcnq1ot1, and miR‐374‐3p was negatively correlated with Fstl1 (Figure 5I,J).

### 2.8 Creb3l1 Positively Regulates the Transcription of lncRNA Kcnq1ot1

**Figure 6:**
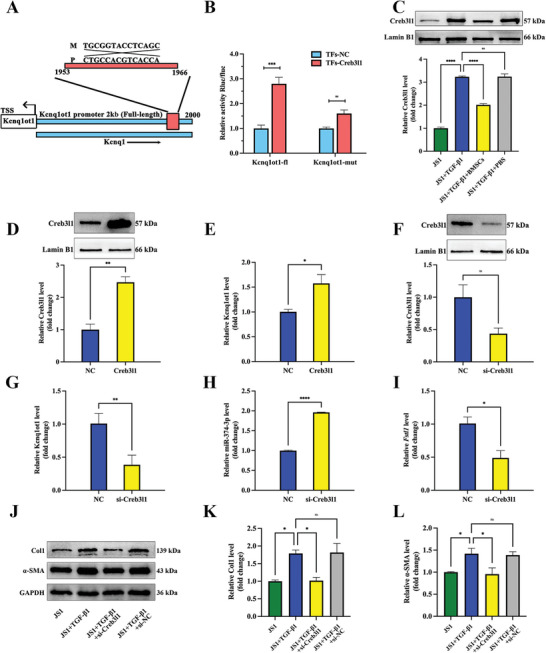
Creb3l1 positively regulates the transcription of lncRNA Kcnq1ot1. A) The positions of full‐length (fl) and fragments of lncRNA Kcnq1ot1 promoter reporters. B) Luciferase activity assay shows the binding relationship between lncRNA Kcnq1ot1 full‐length promoter and Creb3l1. C) Representative western blot bands and statistical results of Creb3l1 in activated JS1 cells by BMSCs treatment. D) Efficiency of Creb3l1 overexpression in JS1 cells. E) Expression of lncRNA Kcnq1ot1 after Creb3l1 overexpression. F) Efficiency of si‐Creb3l1 in JS1 cells. G) Expression of lncRNA Kcnq1ot1 after si‐Creb3l1. H) Expression of miR‐374‐3p after si‐Creb3l1. I) Expression of Fstl1 after si‐Creb3l1. J) Representative western blot bands of Col1 and *α*‐SMA in JS1 cells. K,L) The effect of Creb3l1 knockdown on Col1 (K) and *α*‐SMA (L) expression in activated JS1 cells. *n* = 3 in each group, * *p* < 0.05, ** *p* < 0.01, **** *p* < 0.0001, ns *p* ≥ 0.05.

The binding sites of Creb3l1 and lncRNA Kcnq1ot1 were predicted by UCSC (hppt://genome.ucsc.edu) and JASPAR (http://www.jaspar.genereg.net, **Figure** **6**A, Figure S3A,B, Supporting Information). We constructed a full‐length (fl) lncRNA Kcnq1ot1 promoter (2 kb) and mutant vector (mut) luciferase reporter plasmid at sites 1953–1966 and performed luciferase activity assay. The results showed that RLU was positive for the 2 kb fl and negative for the mut (Figure 6B). This indicates that Creb3l1 is bound to the lncRNA Kcnq1ot1 promoter sequence 1953–1966. BMSCs treatment also decreased the expression of Creb3l1 in mice with liver cirrhosis (Figure 6C). In addition, the expression of Kcnq1ot1 was increased via the overexpression of Creb3l1 (Figure 6D,E), while the expression of Kcnq1ot1 and Fstl1 decreased and miR‐374‐3p increased via si‐Creb3l1 (Figure 6F–I). These results confirm the hypothesis that Creb3l1 positively regulates Kcnq1ot1 transcription. Overall, the above experiments suggest that si‐Kcnq1ot1 can alleviate the activation of JS1 cells. Then, we detected the effect of si‐Creb3l1 on activated JS1 cells and the result showed that *α*‐SMA and Col1 expression level were decreased after the knockdown of Creb3l1 (Figure 6J–L).

### 2.9 LncRNA Kcnq1ot1 Knockdown Alleviates Liver Cirrhosis in Mice by Regulating the miR‐374‐3p/ Fstl1 Signaling Pathway

**Figure 7:**
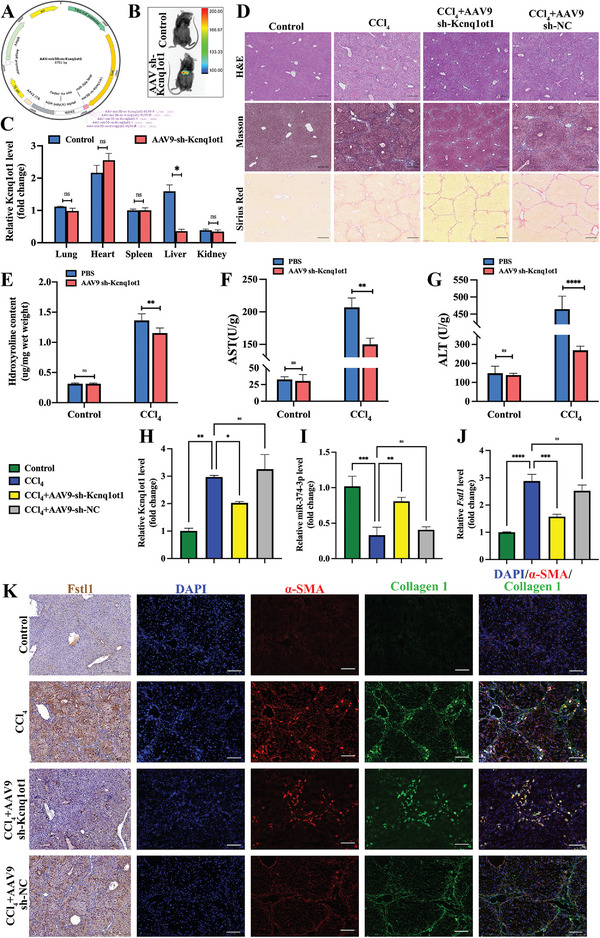
The knockdown of lncRNA Kcnq1ot1 alleviates liver cirrhosis by miR‐374‐3p/Fstl1 in vivo. A) Schematic presentation of the strategy for deletion of lncRNA Kcnq1ot1. B) Fluorescence images of AAV9‐sh‐lncRNA Kcnq1ot1 treated mice. C) LncRNA Kcnq1ot1 expression of different parenchymal organs after AAV9‐sh‐Kcnq1ot1. D) H&E, Masson, and Siris Red staining images. E) Liver hydroxyproline content. F,G) Liver AST (F) and ALT (G) expression level. H,J) LncRNA Kcnq1ot1 (H), miR‐374‐3p (I), and Fstl1 (J) mRNA expression levels in JS1 cells. K) Immunofluorescence images of Col1 and *α*‐SMA in mice liver. *n* = 3 in each group, * *p* < 0.05, ** *p* < 0.01, *** *p* < 0.001, **** *p* < 0.0001, ns *p* ≥ 0.05. Scale bar, 250 µm.

A liver‐specific AAV9 (Adeno‐Associated Virus 9)‐sh‐lncRNA Kcnq1ot1 vector was constructed (**Figure** **7**A), and a cirrhosis model was established to detect the role of Kcnq1ot1 in cirrhosis (Figure S4A, Supporting Information). The AAV9 vector was enriched in the liver tissues (Figure 7B). In addition, we evaluated the changes of Kcnq1ot1 expression in mice parenchymal organs before and after AAV9‐sh‐Kcnq1ot1 transfection. The decrease in Kcnq1ot1 was only observed in liver tissue (Figure 7C). H&E, Masson, and Sirius red pathological staining showed that Kcnq1ot1 knockdown attenuated the progression of cirrhosis mice (Figure 7D). Simultaneously, the immunohistochemical results of CK19 indirectly confirmed that the degree of bile duct hyperplasia was alleviated with Kcnq1ot1 knockdown (Figure S4B, Supporting Information). Additionally, hydroxyproline content was decreased in sh‐Kcnq1ot1 administered liver cirrhosis mice (Figure 7E). The expression of AST and ALT also verified that the knockdown of Kcnq1ot1 could restore liver function in cirrhotic mice (Figure 7F,G). The effect of Kcnq1ot1 knockdown was associated with increased miR‐374‐3p and decreased Fstl1 expression in the liver of cirrhotic mice (Figure 7H–J). In addition, Fstl1, *α*‐SMA, and Col1 protein expression were decreased in cirrhotic mice treated with sh‐Kcnq1ot1 (Figure 7K).

## 3 Discussion

**Figure 8:**
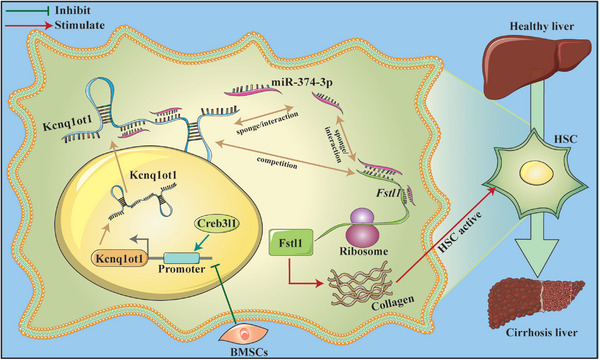
BMSCs alleviate liver cirrhosis through modulating Creb3l1 / lncRNA Kcnq1ot1 / miR‐374‐3p / Fstl1 pathway.

Our study confirmed that BMSCs can alleviate liver cirrhosis in mice. Subsequently, we found that the lncRNA Kcnq1ot1/miR‐374‐3p/Fstl1 signaling pathway is involved in the progression of liver cirrhosis and can be regulated by BMSCs. In addition, Creb3l1 can positively regulate Kcnq1ot1 expression at the transcriptional level (**Figure** **8**).

Stem cells can be used to treat various diseases.^[^ ^19^, ^20^ ^]^ In particular, stem cell therapy for cirrhosis has been used in clinical trials.^[^ ^21^ ^]^ Shiratsuki et al. compared two commonly used mesenchymal stromal cells (MSCs), finding that the risk of pulmonary embolism was higher during adipose‐stromal‐cell than during BMSC treatment. Their findings suggest that BMSCs are a safer cell source for regenerative therapy.^[^ ^5^, ^18^ ^]^ In this study, we selected BMSCs to treat liver cirrhosis and studied their mechanism. Dir‐labeled BMSCs were recruited to the inflammatory site in vivo, and liver function and hydroxyproline expression were reduced after BMSCs treatment. Col1 protein production and activated‐HSC surface marker *α*‐SMA decreased after BMSCs treatment in vitro. BMSCs reduced collagen production by inhibiting HSC activation, thereby alleviating cirrhosis. Recent findings also proved that BMSCs can participate in the treatment of liver cirrhosis by regulating lncRNA.^[^ ^10^ ^]^ In our study, we found for the first time that BMSCs alleviate liver cirrhosis by downregulating lncRNA Kcnq1ot1.

In recent years, the roles of lncRNAs in liver fibrosis have been gradually revealed.^[^ ^22^ ^]^ Studies have shown that lncRNA ScaRNA10 is remarkably upregulated in human and mouse fibrotic livers and could induce hepatocyte apoptosis and HSCs activation.^[^ ^23^ ^]^ LncRNA NEAT1 was also upregulated in liver fibrosis and activated HSCs via sponging of miR‐139‐5p.^[^ ^24^ ^]^ Our previous study found that Kcnq1ot1 is highly expressed in liver cirrhosis.^[^ ^16^ ^]^ The present study showed that BMSCs treatment can downregulate Kcnq1ot1 expression in cirrhotic mouse models both in vivo and in vitro. We then investigated the mechanism of Kcnq1ot1 in cirrhosis. We found that Kcnq1ot1 is mainly distributed in the cytoplasm of JS1 cells. Using starBase 3.0 software, we predicted that miR‐374‐3p can directly bind with lncRNA Kcnq1ot1 and Fstl1. The predicted binding sites were verified by luciferase activity assay. This was in accordance with the RNA sequencing data that miR‐374‐3p is downregulated and Fstl1 is upregulated in mice with liver cirrhosis.^[^ ^16^ ^]^ Fstl1 is a secreted glycoprotein that belongs to the follistatin and SPARC families.^[^ ^25^ ^]^ Studies have shown that Fstl1 contributes to the progression of liver fibrosis.^[^ ^26^ ^]^ Xu et al. found upregulation of Fstl1 in human and mouse fibrotic livers and activated HSCs. The knockdown of Fstl1 could ameliorate HSC activation and extracellular matrix production.^[^ ^27^ ^]^ Zheng et al. also clarified that Fstl1 facilitates the immunosuppressive of MSCs on macrophages and guarantees the anti‐fibrotic effect of MSCs in liver fibrosis.^[^ ^28^ ^]^ Fstl1 is highly expressed in activated HSCs, while Fstl1 knockdown effectively suppresses HSCs proliferation by the TGF‑*β*1/Smad3 signaling pathway.^[^ ^29^ ^]^ In our study, Fstl1 was also highly expressed in cirrhosis and regulated by Kcnq1ot1 and miR‐374‐3p.

We further investigated how BMSCs regulate Kcnq1ot1 expression. Based on the prediction of UCSC and JASPAR, we found that Creb3l1 could bind with the promoter of lncRNA Kcnq1ot1. Creb3l1 is a transcription factor of the CREB/ATF family.^[^ ^30^ ^]^ The cAMP‐responsive element binding protein 3 (CREB3) family members are located in the ER membrane and act as transcription factors when cleaved by S1P and S2P proteases.^[^ ^31^ ^]^ In this study, we found that Creb3l1 directly binds to the 1953–1966 bp promoter region of lncRNA Kcnq1ot1 through luciferase activity assay experiments. Subsequently, we found that lncRNA Kcnq1ot1 is upregulated by Creb3l1 overexpression and downregulated by Creb3l1 inhibition. We then found that Creb3l1 decreases after BMSCs treatment. Miyake et al. demonstrated that Creb3l1 contributes to kidney fibrosis in podocytes.^[^ ^32^ ^]^ In our study, we found that BMSCs treatment may regulate Kcnq1ot1 by downregulating the Creb3l1 expression.

In summary, BMSCs alleviate liver cirrhosis by modulating the Creb3l1/lncRNA Kcnq1ot1/miR‐374‐3p/Fstl1 pathway. Our research offers a novel theoretical framework for the use of BMSCs in the treatment of liver cirrhosis.

## 4 Experimental Section

### Animals

Specific pathogen‐free male BALB/C mice were purchased from the Animal Center of the Second Affiliated Hospital of Harbin Medical University. The mice were housed under a 12 h day per night cycle. Six‐week‐old mice were selected to establish a liver cirrhosis model. The mice were given intraperitoneal injections of 15% carbon tetrachloride (CCl_4_; Macklin, China) dissolved in olive oil thrice weekly at a dose of 1.0 mL kg^−1^ body weight for eight weeks. Then, the model mice were randomly divided into three groups that were given the following treatments in addition to continued thrice weekly intraperitoneal injections of CCl_4_: (1) no additional treatment (*n* = 8); (2) 1 × 10^5^ BMSCs injected into the tail vein twice weekly (*n* = 8); (3) PBS injected into the tail vein twice weekly (*n* = 8). After four weeks, the mice were euthanized, and their liver and other parenchymal organs were harvested. Additionally, six‐week‐old male BALB/C mice were used to isolate BMSCs. The Ethics Committee on the Use and Care of Animals Center of Harbin Medical University approved this study (approval number: SYDW2021‐001).

### Clinical Samples

All patients in this study provided given written informed consent. The patients’ clinical samples were obtained from the Second Affiliated Hospital of Harbin Medical University, including five cases of the normal control group and 10 cases of partial hepatectomy or liver biopsy in patients with cirrhosis. All patients were diagnosed with liver cirrhosis by pathological staining, abdominal ultrasound, and computed tomography. This study was approved by the Medical Ethics Committee of the Second Affiliated Hospital of Harbin Medical University (approval number: KY2022‐283).

### BMSCs Isolation

The male BALB/C mice were sacrificed via cervical dislocation, then drowned in 75% alcohol for 3 min. Next, their skin was carefully cut, and adherent soft tissue was separated. Then, the femurs and tibiae were isolated and subsequently sterilized for 3 min, and a cut was made in the middle of the bone. The bone marrow was then harvested by inserting a 30G size needle at the breakage of the bone and flushing it with DMEM (DMEM, Gibco, USA). A 70 mm nylon mesh filter was used to separate the bone marrow cells. Cells were then plated into 25 cm^2^ plastic cell culture plates at a density of 1 × 10^6^ cells in DMEM containing 20% fetal bovine serum (FBS, ScienCell, USA). Cultures were kept in a 37 °C humidified environment containing 95% air and 5% CO_2_.

### Cell Culture and Cell Activation Induction

Mouse hepatic stellate cell line JS1 cells and human hepatic stellate cell line LX2 cells were purchased from Otwo Biotech Technology Co. Ltd (Shenzhen, China). Both JS1 cells and LX2 cells were stimulated with 5 ng ml^−1^ of TGF‐*β*1 (Peprotech, USA) for 48 h^[^ ^9^, ^33^ ^]^ in DMEM containing 10% FBS (Corning, USA) and kept in a 37 °C humidified environment containing 95% air and 5% CO_2_.

### Transfection Procedure

SiRNA of lncRNA Kcnq1ot1 (sense sequence: 5’‐GAACCUAGAAGCAAAUCCATT‐3’, antisense sequence: 5’‐UGGAUUUGCUUCUAGGUUCTT‐3’); siRNA of Fstl1 (sense sequence: 5’‐GCAAUGACCUGUGAUGGAATT‐3’, antisense sequence: 5’‐UUCCAUCACAGGUCAUUGCTT‐3’); negative control (NC) for siRNA (sense sequence: 5’‐UUCUCCGAACGUGUCACGUTT‐3’, antisense sequence:5’‐ACGUGACACGUUCGGAGAATT‐3’); miR‐374‐3p mimic (sense sequence:5’‐GGUUGUAUUAUCAUUGUCCGAG‐3’, antisense sequence: 5’‐CGGACAAUGAUAAUACAACCUU‐3’); NC for miR‐374‐3p (sense sequence: 5’‐UUCUCCGAACGUGUCACGUTT‐3’, antisense sequence: 5’‐ACGUGACACGUUCGGAGAATT‐3’); X‐tremeGENE siRNA Transfection Reagent was used to transfect siRNA and miRNA for 36 hours.

*The 5‐ethynyl‐2″‐deoxyuridine assay*: A 5‐ethynyl‐2″‐deoxyuridine assay (EdU) solution (ApexBio Technology, USA) was diluted to 10 µM. A total of 100 µL of the EdU solution was then pipetted to each well for 2 h. Following this, DAPI was added to stain the cell nucleus. Stained cells were then imaged under a fluorescence microscope. ImageJ software was used to detect the ratio of EdU‐positive cells was calculated.

### Flow Cytometry

Primary isolated BMSCs were identified via flow cytometry. The specific expressions of antibody CD29 (FITC), CD90 (PE), CD43 (PerCP), and CD31 (APC) (BD Biosciences, USA) were used to identify BMSCs.^[^ ^18^ ^]^ After treatment for 24 h, the third generation of BMSCs was resuspended in 100 µl of a PBS buffer and incubated in antibodies for 30 min in the dark. Data were then analyzed using FlowJo 7.6 software.

### BMSCs Osteogenesis and Adipogenicity

The third generation of BMSCs was cultured to induce osteogenesis and adipogenicity. After 4% paraformaldehyde fixation, Alizarin red and Oil Red O were inserted separately to stain osteogenesis and adipogenicity so as to identify stem cell multilineage differentiation potential.

### Histological Analysis

Fresh liver tissues were fixed in 4% paraformaldehyde, embedded in paraffin, and sectioned. The liver tissues were then sliced into 3 µm‐thick slices and stained with hematoxylin‐eosin (H&E) staining, Masson staining, and Sirius red staining. The results were examined using an optical microscope.

### The Hydroxyproline Assay

The reagent was used to process the liver tissues, and a hydroxyproline assay kit (Njjcbio, China) was used to detect them. Additionally, a microplate reader was used to measure the optical density at 550 nm.

### Liver Function

ALT and AST are 2 routine clinical indicators of liver function, and abnormal ALT and AST levels are related to liver cirrhosis. Therefore, the liver tissues were processed using the reagent and detected using the ALT and AST assay kit (Njjcbio, China). A microplate reader was then used to measure the optical density at 510 nm. ALT and AST levels were calculated according to the formula in the instruction manual.

### Immunohistochemical Staining

The paraffin‐embedded mouse liver was cut into slices with a thickness of 3 µm. After sodium citrate repair treatment, the exposed antigen was combined with the primary anti‐CK19 antibody (ab52625, Abcam, UK) and anti‐Fstl1 antibody (ab223287, Abcam, UK), following which it was color‐rendered with DAB.

### Immunofluorescence Staining

The JS1 cells were seeded onto coverslips, and the treated livers were sectioned with a cryostat (3 µm). Then, the samples were fixed with 4% paraformaldehyde for 20 min, washed with PBS, and permeabilized in PBS containing 0.3% Triton X‐100 for 20 min. The samples were then incubated with an anti‐*α*‐SMA antibody (ab124964, Abcam, UK) and an anti‐Col1 antibody (ab138492, Abcam, UK) overnight at 4 °C, then incubated with a secondary antibody for 1 h in the dark. Notably, nuclei were labeled using Dapi. The results of the immunostaining were examined using a fluorescence microscope, and data were analyzed using ImageJ software. ImageJ software was used to detect the fluorescence intensity of *α*‐SMA and DAPI respectively, and the ratio of the *α*‐SMA‐positive area was calculated.

### Fluorescence in Situ Hybridization

Here, the JS1 cells were seeded onto coverslips, and a lncRNA Kcnq1ot1 probe was added to the hybridization solution for overnight hybridization at 37 °C in the dark. Notably, the nuclei were labeled using Dapi, and the results were photographed using a laser‐scanning confocal microscope.

### Quantitative Reverse Transcription‐PCR

LncRNA and mRNA samples from cultured cells and tissues were extracted using the TRIzol standard protocol (9109, Takara). For each sample, the RNA was converted to 500 ng of cDNA (RR036A, Takara). Additionally, miR‐374‐3p was extracted using the TRIzol standard protocol (9753A, Takara) and converted to 500 ng of cDNA for the qPCR (638 314, Takara). The transcript levels of lncRNA Kcnq1ot1, miR‐374‐3p, Fstl1, Col1, and *α*‐SMA were then detected using the TB Green Premix Real Time PCR system (RR082A, Takara). Finally, U6 and GAPDH were used as internal RNA controls for miRNA and mRNA/lncRNA, respectively.

### Western Blot

The total protein was extracted from cells and liver tissues with a RIPA buffer (Solarbio, Beijing, China) containing 1% phenylmethylsulfonyl fluoride (Beyotime, Shanghai, China). The protein concentration was determined using a bicinchoninic acid kit (Beyotime, Shanghai, China). After boiling it for 10 min, the protein sample was loaded to each lane and separated on 6% and 10% of the SDS‐PAGE. Then, the protein in the gel was transferred onto polyvinylidene fluoride membranes. After blocking these for 15 min, the primary antibody was added. The antibodies against *α*‐SMA (1:4000 dilution), Col1 (1:1000 dilution), and Fstl1 (1:1000 dilution) were incubated overnight at 4 °C. Notably, GAPDH was used as an internal protein control. Finally, Western blot bands were acquired using the Biochem System (BIO‐RAD, USA).

### Bone Marrow Mesenchymal Stromal Cell and JS1 Cells Co‐Culture

BMSCs and JS1 cells were co‐cultured in 24‐well transwell inserts. The lower chambers were seeded with different groups of JS1 cells, with a density of 1 × 10^5^ cells in DMEM containing 10% FBS; the upper chambers were filled with BMSCs with a density of 5 × 10^4^ cells in DMEM. The transwell system was incubated at 37 °C in 5% CO_2_ for 24 h. Then, the JS1 cells were collected from the lower chambers for further detection.

### The Luciferase Reporter Assay

The luciferase reporter gene plasmid and the control plasmid co‐transfected cells after the cell fusion degree reached 70%. Moreover, qPCR was used to amplify the wt Kcnq1ot1 sequence containing miR‐374‐3p binding site. After 36 h of co‐transfection, luciferase activity in the cell lysate was detected using the dual luciferase reporting system (Promega, USA). It is similarly established a wt Fstl1 sequence containing a miR‐374‐3p binding site.

The fl and 1953–1966 mut promoter fragments from the gene transcribed into Kcnq1ot1 were inserted into a dual luciferase gene reporter plasmid. The overexpressed Creb3l1 and NC plasmids were transfected into cells. The groups were as follows: Kcnq1ot1‐fl+TFs‐NC, Kcnq1ot1‐fl+TFs‐Creb3l1, Kcnq1ot1‐mut+TFs‐NC, and Kcnq1ot1‐mut+TFs‐Creb3l1. When the transcription factor Creb3l1 binds to the 1953–1966 site in fl, it can regulate Kcnq1ot1 transcription and fluorescence intensity. Fluorescence values were measured by fluorescence luminescence (BioTek, USA).

### BMSCs Tracing In Vivo and Mice Imaging In Vivo

To track the organ distribution of BMSCs in cirrhotic mice, BMSCs cell membranes were stained with lipophilic carbon cyanine fluorescent dye DiR (Invitrogen, USA) according to the manufacturer's instructions. The third generation of BMSCs was purified using PBS and centrifuged to collect cells, which were incubated with DiR at a final concentration of 10 mM at room temperature for 30 min. BMSCs labeled with DiR were transplanted through the tail vein of mice. After 48 h, the distribution of BMSCs labeled with DiR in mice was detected using in vivo imaging system (IVIS, Night OWL II LB983, Germany). After euthanasia, the distribution of BMSCs labeled with DiR in various parenchymal organs (liver, spleen, pancreas, heart, lung, and kidney) of mice was detected. Bruker MI SE software was used to analyze the captured images.

### AAV9 sh‐Kcnq1ot1 Transfected Mice

Four‐week‐old mice were injected with AAV9‐tgb (Hanbio, China) through their tail vein to establish lncRNA Kcnq1ot1 knockdown mice. The cirrhosis model was established two weeks after transfection. After 12 weeks of cirrhosis model establishment, the transfection was detected in mice using an imaging system before they were euthanized, and their liver and various parenchymal organs were collected for detection.

### Statistical Analysis

Data were expressed as mean ± SEM. A one‐way analysis of variance was used to analyze multiple group comparisons, and two‐tailed unpaired Student's *t*‐test was used to analyze two‐group comparisons. Here, *p* < 0.05 was considered a statistically significant difference. GraphPad 9.0 was used to analyze the data. Each experiment was repeated three times independently, and all data were included.

## Conflict of Interest

The authors declare no conflict of interest.

## Author Contributions

H.Z. and Y.J. contributed equally to this work. S.J., Y.J., and H.Z. vised the study design, main conceptual ideas, and project outline; H.Z. contributed to cell experiments; Y.G. and H.Z. draw mechanism diagrams; S.L., H.Z. and R.X. contributed to animal experiments; Y.J., J.S., J.L., Y.H. and X.Z. processed and analyzed the data; H.Z., X.Z., L.Z., J.Q. and X.G. procured human samples; H.Z. and Y.J. wrote the manuscript.

## Supporting information

Supporting Information

## Data Availability

The data that support the findings of this study are available from the corresponding author upon reasonable request.
