# Supplementary material for: From Phenomenon to Essence: A Newly Involved lncRNA Kcnq1ot1 Protective Mechanism of Bone Marrow Mesenchymal Stromal Cells in Liver Cirrhosis
Source: Adv Sci (Weinh). 2023 Jun 6;10(21):2206758. doi: 10.1002/advs.202206758 (PMC10375186; doi:10.1002/advs.202206758)
Supplement: Supplementary file 1 — Supporting Information [file ADVS-10-2206758-s001.pdf]

ADVANCED SCIENCE
Open Access

Supporting Information

for *Adv. Sci.*, DOI 10.1002/advs.202206758

From Phenomenon to Essence: A Newly Involved lncRNA Kcnq1ot1 Protective Mechanism of Bone Marrow Mesenchymal Stromal Cells in Liver Cirrhosis

*Hanjing Zhangdi, Yanan Jiang, Yang Gao, Shuang Li, Ruiling Xu, Jing Shao, Jingyang Liu, Ying Hu, Xu Zhang, Xiaoyu Zhang, Lei Zhao, Jihan Qi, Xinyu Geng and Shizhu Jin**

pSupplementary Materials

Supplementary Figure 1. Extraction and identification of BMSCs. A)Schedule of liver cirrhosis model with BMSCs treatment. B) Schematic diagram of mice BMSCs extraction. C) CD29 and CD90 are positive and CD31 and CD43 are negative in BMSCs by flow cytometry. D) Osteogenetic and adipogenic differentiation potential of BMSC. Arrows indicate calcium nodules and lipid droplets. E) *In vivo* imaging fluorescence of mice and parenchymal organs.

Supplementary Figure 2. Therapeutic effect of BMSCs on liver cirrhosis. A) CK19 immunohistochemistry showed bile duct hyperplasia degree in cirrhosis. B) Immunofluorescence images of Col1 and α-SMA in mice liver.

Supplementary Figure 3. Bioinformatics analysis predicts the transcriptional regulatory relationship between Creb3l1 and Kcnq1ot1. A) The combined prediction results of UCSC Genomic Broswer and JASPAR show that Creb3l1 is a transcription factor that positively regulates Kcnq1ot1 transcription (Score = 752; P value <10-7). B) The JASPAR prediction results show that the most likely binding site between Creb3l1 and Kcnq1ot1 Promoter sequence is 1953bp-1966bp (Score= 21.115026; Relative score=0.979)

Supplementary Figure 4. Knockdown Kcnq1ot1 relieved mice liver cirrhosis. A) Schedule of liver cirrhosis model with AAV9-sh-Kcnq1ot1 transfection. B) CK19 immunohistochemistry showed bile duct hyperplasia degree in cirrhosis.

Supplementary Table 1. RNA primer sequences.

Supplementary Table 1.

**Primer**	**Primer sequence (3'-5')**
Human Kcnq1ot1-F	CCAGGCACTTGACACAGCAGAG
Human Kcnq1ot1-R	GAAGGCACAGCAGGGACAATCG
Human Fstl1-F	CGCTGAAGTGGAGAAGATGC
Human Fstl1-R	ACCCAGACAGAGGAGGAGAT
Human miR-374-3p-F	GGCCGCTTAGCAGGTTGTATTATCATT
Human GAPDH-F	CAGGAGGCATTGCTGATGAT
Human GAPDH-R	GAAGGCTGGGGCTCATTT
Mouse Kcnq1ot1-F	AACACTCTGCTGCTTGCCTGAC
Mouse Kcnq1ot1-R	TCCTTGTCGGTCCTGTAGCCATC
Mouse Fstl1-F	TGAGGTAGGTCTTGCCATTACTG
Mouse Fstl1-R	TCTGTGCCAATGTGTTTTGTG
Mouse miR-374-3p-F	CCGCGGTTGTATTATCATTGTCCGAG
Mouse Creb3l1-F	GAAACCCTAGAGACTGCCAAC
Mouse Creb3l1-R	ACCAGAACGAAGCACAAGG
Mouse GAPDH-F	CAAGAAGGTGGTGAAGCAGG
Mouse GAPDH-R	CCACCCTGTTGCTGTAGCC
Mouse α-SMA-F	GTGAAGAGGAAGACAGCACAG
Mouse α-SMA-R	GCCCATTCCAACCATTACTCC
Mouse Col1-F	ATCTCCTGGTGCTGATGGAC
Mouse Col1-R	ACCTTGTTTGCCAGGTTCAC
U6-F	GGAACGATACAGAGAAGATTAGC
U6-R	TGGAACGCTTCACGAATTTGCG
